# Inpatient use and area-level socio-environmental factors in people with psychosis

**DOI:** 10.1007/s00127-018-1534-x

**Published:** 2018-05-23

**Authors:** Margaret Heslin, Mizanur Khondoker, Hitesh Shetty, Megan Pritchard, Peter B. Jones, David Osborn, James B. Kirkbride, Angus Roberts, Robert Stewart

**Affiliations:** 1 Institute of Psychiatry, Psychology and Neuroscience, Kings College London, London, UK; 2 Norwich Medical School, University of East Anglia, Norwich, UK; 3 South London and Maudsley NHS Foundation Trust, London, UK; 4 University of Cambridge, Cambridgeshire and Peterborough NHS Foundation Trust, Cambridge, UK; 5 UCL, Camden and Islington NHS Foundation Trust, London, UK; 6 UCL, Division of Psychiatry, London, W1T 7NF UK

**Keywords:** Psychoses, Socio-environmental factors, Outcomes, Deprivation

## Abstract

**Purpose:**

There is consistent evidence that socio-environmental factors measured at an area-level, such as ethnic density, urban environment and deprivation are associated with psychosis risk. However, whether area-level socio-environmental factors are associated with outcomes following psychosis onset is less clear. This study aimed to examine whether the number of inpatient days used by people presenting to mental health services for psychosis was associated with five key area-level socio-environmental factors: deprivation, ethnic density, social capital, population density and social fragmentation.

**Methods:**

Using a historical cohort design based on electronic health records from the South London and Maudsley NHS Trust Foundation electronic Patient Journey System, people who presented for the first time to SLAM between 2007 and 2010 with psychosis were included. Structured data were extracted on age at presentation, gender, ethnicity, residential area at first presentation and number of inpatient days over 5 years of follow-up. Data on area-level socio-environmental factors taken from published sources were linked to participants’ residential addresses. The relationship between the number of inpatient days and each socio-environmental factor was investigated in univariate negative binomial regression models with time in contact with services treated as an offset variable.

**Results:**

A total of 2147 people had full data on area level outcomes and baseline demographics, thus, could be included in the full analysis. No area-level socio-environmental factors were associated with inpatient days.

**Conclusion:**

Although a robust association exists between socio-environmental factors and psychosis risk, in this study we found no evidence that neighbourhood deprivation was linked to future inpatient admissions following the onset of psychosis. Future work on the influence of area-level socio-environmental factors on outcome should examine more nuanced outcomes, e.g. recovery, symptom trajectory, and should account for key methodological challenges, e.g. accounting for changes in address.

**Electronic supplementary material:**

The online version of this article (10.1007/s00127-018-1534-x) contains supplementary material, which is available to authorized users.

## Introduction

Psychotic disorders, including schizophrenia and related disorders, bipolar disorder and depressive psychosis, are often long-standing with substantial impacts on individuals across the life course [1]. They are ranked fifth and sixth in men and women, respectively, as a cause of years lived with disability, comprising a moderate to high proportion of the global burden of disease [2]. However, outcomes in people with psychosis show high individual variability, from discrete episodes followed by prolonged recovery in some people, to a chronic and highly disabling course in others [3]. As well as the impact on the individual and their family, psychosis outcomes are of enormous importance to mental health services with substantial resource implications at local and national levels [4].

Examining factors predicting outcomes in people with psychosis is important because this potentially both advances our understanding of underlying aetiology and provides potential targets for interventions [5]. Despite the importance and variability of symptomatic, functional and service use outcome in psychosis, little is known about environmental predictors of prognosis following the onset of the disorder. There is consistent evidence that socio-environmental factors measured at an area level (e.g. neighbourhood) are associated with the risk of psychosis and psychotic symptoms. This includes a protective effect of ethnic density [6], urban environment [7] and deprivation [8–10]. However, whether area level socio-environmental factors are associated with outcomes following onset of psychosis is less clear.

We know that following psychotic disorders, up to one-third of people will change residential location within 6 years of onset to both more and less deprived areas [11]. What remains unclear, however, is whether people with psychotic disorder exposed to more adverse social environments experience worse subsequent health outcomes (for example, higher readmission rates), Limited evidence suggests that area-level deprivation is associated with higher risk of inpatient admission for mental disorders in general [12], for psychosis specifically [8], and for both non-affective and affective disorders independently [13]. However, these studies made no adjustment for other area-level socio-environmental factors such as ethnic density, social capital, or population density which could also influence such associations.

Although it is a crude measure, inpatient use can be used as a proxy for mental health service utilisation which in turn can be used as a proxy for disease activity in the context of psychotic disorders [14, 15]. Thus, the aim of this study was to examine whether the number of inpatient days following initial presentation to mental health services for psychosis was associated with five key area-level socio-environmental factors: deprivation, ethnic density, social capital, population density and social fragmentation.

## Methods

### Design

This study used a historical cohort design based on Electronic Health Records (EHRs) from the South London and Maudsley (SLaM) NHS Trust Foundation electronic Patient Journey System (ePJS). Since 2006, comprehensive health records from over 280,000 patients in the ePJS have been de-identified and made accessible via the Clinical Record Interactive Search tool (CRIS). CRIS holds all information documented by professionals involved in the provision of specialist mental health care for all people in contact with SLaM mental healthcare services from 1 January 2007 to date [16]. SLaM covers the four London boroughs of Lambeth, Southwark, Lewisham and Croydon.

### Sample

The sample comprised all cases age 16 and over who presented for the first time to SLaM between 1 January 2007 and 31 December 2010 with a diagnosis of any psychotic disorder (ICD-10 codes F20-29, 30.2, 31.2, 31.5, 32.3, and 33.3 [17]). Diagnostic information was drawn both from structured fields in the record and a natural language processing algorithm extracting diagnostic statements in text fields [16]. The natural language processing algorithm simply sought to extract any text strings associated with a diagnosis statement to supplement the existing structured fields. Individuals were followed from the date of their first diagnosis for up to 5 years.

We excluded participants without a postcode at first presentation within the four-borough catchment. This was carried out to exclude people who lived in areas covered by neighbouring mental health service providers, but who may have presented to SLaM emergency services, or to exclude referrals from outside of the catchment to specialist national services provided by SLaM. Participants of no fixed abode were also excluded by design.

### Measures

Structured data were extracted on age at presentation, gender, ethnicity, education level, employment status and occupation and postcode linked in CRIS to two administrative geographical levels: Lower (LSOA) and Middle Super Output Areas (MSOA). These geographical units were used to link participants to several socio-environmental indices (see below). LSOAs and MSOAs are geographical areas made up of clusters of socially homogenous postcodes. LSOAs have an average of average 1500 residents (minimum 1000 residents) while MSOAs have an average of 7200 residents (minimum 5000 residents) [18]. Data on employment status, occupation, highest educational level and age at leaving education were only available for the following percentages of people: 32, 31, 51, and 20%. Due to the poor availability of these data, they were excluded from the analyses. Ethnicity was recorded in patient records according to the 16 + 1 ethnic data categories defined in the 2001 census. The outcomes, number of inpatient days over a 1- and 5-year follow-up from the first contact with mental health services, were calculated from ward stay tables, routinely recorded within CRIS, which contain the date of admissions and discharge from inpatient wards. The total time with services was calculated as the time between the first contact and last contact within the 5-year window.

To classify neighbourhood socioenvironmental exposure to deprivation, we used the English Indices of deprivation. These routinely collected national indicators were based on 2007 data, temporally the closest to the baseline date for most participants. Indices were provided at the LSOA level [19]. In our analyses, we assigned participants to their Index of Multiple Deprivation score for their residential address at first presentation.

Population density was determined using 2011 census data (temporally the closest to the baseline date) on the number of people per hectare [20], and based on LSOA11 codes. Ethnic density was defined as the proportion of people from the same ethnic group as the participant living in their LSOA [6], also estimated from the ONS [21] 2011 census. Data on social capital were determined using voter turnout at local government elections as a proxy [9]; however, these data were not available at the LSOA level, thus boroughs had to be used. LSOA for each person was mapped to borough to link these data, and data on the percentage voter turnout were taken from the 2009 European Parliament election [22] as this was temporally the closest to the period of study.

Social fragmentation was calculated for each LSOA from four measures of social composition in the 2011 census: unmarried adults [23], single-person households [24], households privately renting [25], and population turnover (2009–2010 data) [26]. Consistent with previous studies, we summed the *z* scores (number of standard deviations above/below the population mean when the distribution is normal) to create a social fragmentation index [27, 28]. Population turnover was calculated as the sum of in- and out-migration in the 12 months prior to the 2011 census [29] and was only available at the MSOA level so was calculated using MSOA11 codes.

### Ethics

Ethical approval as an anonymised database for secondary analysis was originally granted in 2008 and renewed for a further 5 years in 2013 (Oxford C Research Ethics Committee, reference 08/H0606/71 + 5). The study presented in this paper has been approved by the CRIS Oversight Committee.

### Analysis

Data were first described using mean values, standard deviations and ranges, or frequencies and percentages as appropriate. The relationships between area-level factors were investigated using correlations. The relationship between the number of inpatient days and each socio-environmental factor was investigated in univariate negative binomial regression models with time in contact with services treated as an offset variable. Negative binomial modelling was preferred over more typical Poisson regression for the type of outcome (count data with positively skewed distribution) to account for over-dispersion in the data. Regression analyses were conducted with adjustment for age, gender and BME status. STATA 14 [30] was used for the analyses.

### Sensitivity analyses

We conducted sensitivity analysis regarding the follow-up period since lack of contact might occur for a range of reasons: the person is still living in the catchment area but is well and not in need of contact with services; or the person has left the catchment area but is unwell and being treated by another service. To address this, a number of sensitivity analyses were conducted, as follows:

- Reanalysis of inpatient days over one year based on the full sample (less reliant on five year data) controlling for length of time in contact with services;
- Reanalysis of inpatient days over 5 years based on those in contact with services for the full 5 years only (excludes those who might have left the area but probably sampling a more severely ill population);
- Reanalysis of inpatient days over 1 year based on those in contact with services for the 1 year only (excludes those who might have left the area but probably sampling a more severely ill population).

## Results

### Descriptive analysis

A total of 3292 people presented to SLAM services for the first time between 1 January 2007 and 31 December 2010 and received a psychosis diagnosis. Of these 3292 cases, 792 (24.1%) were out of area referrals and 322 (9.8%) had missing postcode information so were excluded from the analyses. This left a total sample of 2272 patients, of which 2147 patients had full data on area-level outcomes, age, gender and ethnicity so could be included in the full analysis.

Table 1 describes the demographics of the full sample. The mean age of the sample at first presentation was 42 years (standard deviation 18 years). Of the 2147, 50.6% (*n* = 1087) were male. The largest ethnic group was White British with 29.4% of the sample (*n* = 632) followed by 20.0% Black African (*n* = 430) and 10.6% Black other. The number of days under the care of SLAM services was skewed with a median of 601 (i.e. 1.6 years) (interquartile range 57–1766 days) with a range from 1 day to the maximum 5 years. 23.3% (*n* = 501) of cases were under the care of SLAM services for the full 5 years follow-up, while 57.8% (*n* = 1241) were with services for at least one year.

**Table 1:** Basic demographics of the sample

Demographic variable	Mean (SD)
Age	42.17 (18.23)
	Number (%)
Gender	
Male	1087 (50.63)
Female	1060 (49.37)
Ethnicity	
White: English/Welsh/Scottish/Northern Irish/British	632 (29.44)
Black/African/Caribbean/Black British: Africa	430 (20.03)
Black/African/Caribbean/Black British: Other Black	228 (10.62)
Black/African/Caribbean/Black British: Caribbean	209 (9.73)
White: other	201 (9.36)
Any other ethnic group	161 (7.50)
Asian/Asian British: other Asian	95 (4.42)
White: Irish	48 (2.24)
Asian/Asian British: Indian	32 (1.49)
Asian/Asian British: Pakistani	25 (1.16)
Asian/Asian British: Chinese	21 (0.98)
Mixed/multiple ethnic group: White and Black Caribbean	20 (0.93)
Asian/Asian British: Bangladeshi	14 (0.65)
Mixed/multiple ethnic group: White and Black African	12 (0.56)
Mixed/multiple ethnic group: Other Mixed	12 (0.56)
Mixed/multiple ethnic group: Mixed White and Asian	7 (0.33)

The number of days as an inpatient ranged between 0 and 1777 with a median of 6 days and interquartile range of 0–69 days. The distribution of inpatient days was positively skewed, with many cases having few inpatient days and few cases having many inpatient days.

Table 2 describes area-level socio-environmental factors in the SLaM catchment area compared to London and English normative data. There was considerable heterogeneity between areas in all area level factors shown by the wide sample ranges and standard deviations. These ranges are similar to the ranges for London and England overall. In terms of deprivation, the table shows that the socio-environmental factor means for the study sample are similar to the mean for England although the deprivation score was higher in the study sample indicating worse deprivation. However, ethnic density, population density and social fragmentation are substantially higher on average in the study location sample compared to that for England overall.

**Table 2:** Area-level socio-environmental factors scores in the area sampled from compared to the whole of England and London

Area level factor	Sample mean (SD)	Sample range	Mean for London (SD)	Range for London	Mean for England (SD)	Range for England
Deprivation	28.28 (10.29)	3.93 to 60.58^a^	25.24 (13.24)	1.70 to 66.21^a^	21.67 (15.51)	0.53 to 87.80^#^
Minority Ethnic group density (percentage of minority Ethnic people living within each area)	57.42 (16.31)	12.65 to 88.55	54.12 (21.49)	3.39 to 98.86	19.26 (22.23)	0.28 to 99.37
Population Density Score (number of people per hectare)	104.54 (55.06)	4.50 to 344.60	95.94 (61.22)	1.20 to 684.70^b^	42.63 (42.26)	0.00 to 684.70^##^
Social capital	31.22 (1.57)	30.15 to 33.54	33.55 (3.26)	27.27 to 41.72	36.73 (5.20)	20.77 to 54.94
Social fragmentation	2.97 (2.33)	− 2.94 to 10.54	2.24 (2.52)	− 3.25 to 10.93	− 0.08 (2.62)	− 4.21 to 17.18

^a^Higher scores indicated worse deprivation

^b^Higher scores indicated more highly populated

There were significant correlations between all of the socio-environmental factors (Table 3). Overall deprivation positively correlated population density (*r* 0.35, *p* < 0.001) and social fragmentation (*r* 0.20, *p* < 0.001), and negatively correlated with ethnic density (*r* − 0.21, *p* < 0.001) and social capital (*r* − 0.32, *p* < 0.01). Ethnic density also positively associated with social capital (0.14, *p* < 0.001), but negatively associated with both population density (− 0.18, *p* < 0.001) and social fragmentation (− 0.19, *p* < 0.001). Population density negatively associated with social capital (− 0.43, *p* < 0.001), but positively associated with social fragmentation (0.33, *p* < 0.001), and social capital and social fragmentation were negatively associated (− 0.44, *p* < 0.001).

**Table 3:** Correlation matrix for area-level socio-environmental factors

Area level factor	Overall IMDS	Ethnic density	Population density	Social capital	Social fragmentation
Deprivation	1.00				
Ethnic density	− 0.21***	1.00			
Population density	0.35***	− 0.18***	1.00		
Social capital	− 0.32***	0.14***	− 0.43***	1.00	
Social fragmentation	0.20***	− 0.19***	0.33***	− 0.44***	1.00

*p < 0.05; ** *p* < 0.01; *** *p* < 0.001

### Regression analysis

Table 4 shows the negative binomial regression models examining the relationship between inpatient days over 5 years and area-level socio-environmental factors. Inpatient days were negatively correlated with age (coefficient: − 0.02, 95% CI − 0.03 to − 0.01, *p* < 0.001). No other variables were associated with inpatient days including all area-level socio-environmental factors.

**Table 4:** Results from the negative binomial regression of inpatient days on area-level socio-environmental factors over 5 years (*n* = 2147)

	Model adjusted for length time with service	Model adjusted for length time with service, age, gender and BME status
	Coefficient (95% confidence interval)	Coefficient (95% confidence interval)
Demographics		
Age	− 0.02 (− 0.03 to − 0.01)***	-
Gender		
Female	–	–
Male	0.22 (− 0.10 to 0.54)	–
BME status		
White British	–	–
BME	0.13 (− 0.22 to 0.48)	–
Area-level socio-environmental factors		
Deprivation	0.00 (− 0.02 to 0.02)	− 0.00 (− 0.02 to 0.02)
Ethnic density	0.16 (− 0.69 to 1.01)	0.59 (− 0.80 to 1.97)
Population density	− 0.00 (− 0.00 to 0.00)	− 0.00 (− 0.00 to 0.00)
Social capital	0.09 (− 0.03 to 0.20)	0.06 (− 0.06 to 0.18)
Social fragmentation	− 0.02 (− 0.11 to 0.07)	− 0.00 (− 0.09 to 0.08)

*p < 0.05; ** *p* < 0.01; *** *p* < 0.001

Sensitivity analyses (see online supplement) showed a very similar picture with age being negatively associated with inpatient days on two out of three of the sensitivity analyses, and no area-level socio-environmental factors being associated with inpatient days at all.

## Discussion

Using data from an electronic health record system and linking with area-level census-derived information, we investigated the link between socio-environmental factors and inpatient use in a cohort of people with psychosis at their first contact with secondary mental health services.

In all but one analysis, being of lower age was associated with more days as an inpatient. In all analyses, no area-level socio-environmental factors were associated with inpatient days. Younger age is often found to be associated with higher mental health service costs [31] so it is unsurprising that it is also associated with inpatient days, as inpatient days are the main driver of overall cost [4].

The lack of associations between area-level socio-environmental factors and inpatient days could reflect a lack of association or could be explained by methodological issues. Censoring follow-up time to the last contact with services, could artificially decrease follow-up time and therefore the proportion of inpatient days to time, biasing the results to those most unwell. The sensitivity analyses aimed to account for the limitation that we were not able to account for changes in address during the follow-up period and only used area level factors linked to postcode at baseline. The sample here is a highly mobile population in an area of high population mobility. It is likely that over 5 years (and even over the 1 year follow-up) people are moving address, and not accounting for this could have confounded the results. Further, area-level socio-environmental factors could impact other outcomes, but inpatient use may be too crude and a measure of outcome which is influenced by other factors such as number of inpatient beds available in the area, perceived risk or patients moving out of the catchment area.

There are a number of other potential limitations. First there is the question of sample selection. Cases were generated based on first contact with mental health services in the catchment area if they also had a psychosis diagnosis ever in the notes. Although all cases had a diagnosis of psychosis at some point, this may not have been the diagnosis at the time in contact with services, or even the latest diagnosis. Some cases may have had other primary diagnoses and generalisability cannot be assumed to all psychosis. This is particularly challenging as diagnoses and presentations in psychiatry frequently change over time [32]. Additionally, the high average age of presenting patients combined with the near even sex ratio in the cohort indicates that this is unlikely to represent a true first episode psychosis cohort. Further, the natural language processing algorithm which was used to extract diagnoses from text fields, although it has been widely used, its accuracy in psychosis has not been evaluated. Second, although each person was selected on their first contact with mental health services in the catchment area, it is possible that some of these people had been treated in other NHS trusts and moved to the catchment area, so first episode psychosis cannot be assumed in all cases; however, with a sample of almost 2000 cases, it was not feasible to examine full records to determine true first contact. Third, social capital was measured using voter turnout at local government elections which was only available at a borough level. As there were only four boroughs, there was not much variation and small area levels could not be examined. Thus, the measure of social capital is very limited. This may have led to skewed results on associations between social capital and inpatient days. Further, the calculation of time with mental health services, which tried to account for people who might have left the area, was calculated based on the first contact and last contact with services, and thus did not account for time when each person had been discharged from the service and were taken on again. These times could represent when patients move out of the catchment area and are being treated by other NHS trusts. Additionally, with the number of analyses conducted, it is likely there are some associations as a result of multiple testing.

An additional limitation around measures relates to the exposure and outcome temporality. The ‘baseline’ time point for the sample varies as people presented at different times. In this sample, the baseline ranges from 1 January 2007 to 31 December 2010. The exposures variables are measured at 2007/8 for social deprivation, 2009 for social capital and 2011 for all other variables. This is because these data are only collected in national audits and so are only available at certain times. This difference in when the exposures are measured and when the outcome is started to be measured from could lead to inaccurate results, however, the exposures are the closest chronologically to the baseline period that are available, and it is not likely that relative characteristics of areas will have changed substantially over the relatively small time period captured.

Finally, the influence of treatment on outcome has not been accounted for here. The aim of treatment is to influence the course of illness in a favourable direction: to reduce symptoms, but also to reduce the need for inpatient or acute treatment, improve functioning and quality of life. This is arguably one of the biggest influences on outcome and needs to be accounted for in any research on outcomes. However, this still tells us whether presentation of social circumstances predict outcomes regardless of treatment.

Future studies should: account for changes in address within the catchment area over time; account for people leaving the catchment area over time; overcome issues with unstable diagnoses; overcome issues around selecting a sample of people with a first contact for a first episode of psychosis; account for treatment. Further, replication of these findings in other samples would be useful and expanding beyond inpatient use to other outcomes, for example, recovery, symptoms trajectory, and mortality.

## Electronic supplementary material

Below is the link to the electronic supplementary material.

Supplementary material 1 (DOCX 15 KB)
