# Supplementary material for: Inpatient use and area-level socio-environmental factors in people with psychosis
Source: Soc Psychiatry Psychiatr Epidemiol. 2018 May 23;53(10):1133–40. doi: 10.1007/s00127-018-1534-x (PMC6182500; doi:10.1007/s00127-018-1534-x)
Supplement: Supplementary file 1 — Supplementary material 1 (DOCX 15 KB) [file 127_2018_1534_MOESM1_ESM.docx]

**Online appendix**

**Table: Results from the negative binomial regression of inpatient days on area-level socio-environmental factors over one year (n=2147)**

|  | ***Model adjusted for length time with service*** | ***Model adjusted for length time with service, age, gender and BME status*** |
| --- | --- | --- |
|  | ***Coefficient (CI)*** | ***Coefficient (CI)*** |
| **Demographics** |  |  |
| Age | -0.01 (-0.02 to -0.00)** | - |
| Gender:  Female  Male | -  0.02 (-0.32 to 0.35) | -  - |
| BME status:  White British  BME | -  0.30 (-0.07 to 0.66) | -  - |
| **Area-level socio-environmental factors** |  |  |
| Deprivation | 0.01 (-0.01 to 0.03) | 0.00 (-0.02 to 0.03) |
| Ethnic Density | -0.64 (-1.57 to 0.29) | -0.22 (-1.64 to 1.91) |
| Population Density | 0.00 (-0.00 to 0.00) | 0.00 (-0.00 to 0.00) |
| Social Capital | 0.02 (-0.10 to 0.15) | 0.03 (-0.09 to 0.16) |
| Social Fragmentation | 0.02 (-0.07 to 0.11) | 0.00 (-0.09 to 0.10) |

*p<0.05; **p<0.01; ***p<0.001

**Table: Results from the negative binomial regression of five year inpatient days for those in contact for the full five years only (n=501)**

|  | ***Model adjusted for length time with service*** | ***Model adjusted for length time with service, age, gender and BME status*** |
| --- | --- | --- |
|  | ***Coefficient (CI)*** | ***Coefficient (CI)*** |
| **Demographics** |  |  |
| Age | -0.02 (-0.03 to -0.00)* | - |
| Gender:  Female  Male | -  0.21 (-0.20 to 0.61) | -  - |
| BME status:  White British  BME | -  0.30 (-0.16 to 0.76) | -  - |
| **Area-level socio-environmental factors** |  |  |
| Deprivation | 0.01 (-0.01 to 0.03) | -0.00 (-0.03 to 0.02) |
| Ethnic Density | -0.73 (-1.80 to 0.35) | 0.38 (-1.52 to 2.28) |
| Population Density | 0.00 (-0.00 to 0.01) | 0.00 (-0.00 to 0.01) |
| Social Capital | -0.03 (-0.17 to 0.11) | -0.01 (-0.16 to 0.13) |
| Social Fragmentation | 0.06 (-0.05 to 0.16) | 0.03 (-0.08 to 0.13) |

*p<0.05; **p<0.01; ***p<0.001

**Table: Results from the negative binomial regression of inpatient days one year inpatient days for those in contact for the full one year only (n1241)**

|  | ***Model adjusted for length time with service*** | ***Model adjusted for length time with service, age, gender and BME status*** |
| --- | --- | --- |
|  | ***Coefficient (CI)*** | ***Coefficient (CI)*** |
| **Demographics** |  |  |
| Age | 0.00 (-0.01 to 0.01) | - |
| Gender:  Female  Male | -  -0.05 (-0.41 to 0.31) | -  - |
| BME status:  White British  BME | -  0.18 (-0.21 to 0.57) | -  - |
| **Area-level socio-environmental factors** |  |  |
| Deprivation | 0.01 (-0.010 to 0.02) | 0.00 (-0.01 to 0.02) |
| Ethnic Density | -0.18 (-1.10 to -0.74) | 0.58 (-1.17 to 2.34) |
| Population Density | 0.00 (-0.00 to 0.00) | 0.00 (-0.00 to 0.00) |
| Social Capital | -0.09 (-0.22 to 0.04) | -0.09 (-0.22 to -0.04) |
| Social Fragmentation | 0.05 (-0.04 to 0.13) | 0.05 -(0.04 to 0.14) |

*p<0.05; **p<0.01; ***p<0.001
